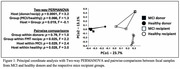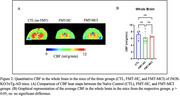# iNOS knockout preserved brain perfusion despite MCI‐induced gut dysbiosis in an Alzheimer's mouse model

**DOI:** 10.1002/alz70855_105884

**Published:** 2025-12-24

**Authors:** Maalavika Govindarajan, Chetan Aware, Kira Ivanich, Ishan Pathak, Yijuan Zhu, Priti Balchandani, Daniel Davis, Aaron Ericsson, Lixin Ma, Ai‐Ling Lin

**Affiliations:** ^1^ University of Missouri, Columbia, MO, USA; ^2^ Icahn School of Medicine at Mount Sinai, New York, NY, USA

## Abstract

**Background:**

Inducible Nitric Oxide Synthase (iNOS) is implicated in exacerbating Alzheimer's Disease (AD) mechanisms. The relationship between imbalanced gut microbiota composition (dysbiosis) and AD pathology is well characterized. Many gut bacteria, including *E. Coli* induce iNOS production, potentially contributing to AD development. To investigate the antagonistic role of iNOS, we created a novel iNOS knockout (iNOS‐KO) mouse model using the 3xTg‐AD mouse model background and performed fecal microbiome transplantation (FMT) to iNOS‐KO/3xTg‐AD mice from mild cognitive impairment (MCI) patients and age‐matched healthy controls (HC). We aim to determine, whether iNOS‐KO can protect cerebral blood flow (CBF), an early marker of AD progression, despite dysbiosis induced by FMT from MCI donors.

**Method:**

Stool samples from MCI patients (*n* =  3) and HC (*n* =  3) (aged 55‐80) were used for FMT in 4‐month‐old iNOS‐KO/3xTg‐AD mice (FMT‐MCI, *n* = 4 and FMT‐HC, *n* = 6) for three consecutive days after a 7‐day antibiotic treatment. Mice without FMT (CTL, *n* = 8) served as naive controls. Four weeks post‐FMT, mouse fecal samples and corresponding donor samples were analyzed using 16S rRNA metagenomic sequencing. Global CBF was measured in a subset of mice (*n* = 4/group) using 7T MRI with Continuous Arterial Spin Labelling (CASL) ‐ Echo Planar Imaging (EPI) sequence.

**Result:**

Beta diversity analysis revealed that the significant microbial diversity observed in MCI and HC donors was imprinted in their respective FMT‐MCI and FMT‐HC recipient mice, indicating a strong donor‐derived microbial signature (Figure 1). FMT‐MCI mice showed increased levels of pathobiont Gram‐positive bacteria (*Clostridium bolteae, Sellimonas intestinalis*) when compared to FMT‐HC mice indicating higher dysbiosis. Despite FMT induced dysbiosis, CBF levels (Figure 2) across the three groups were comparable to each other, attributable to the effect of the iNOS knockout.

**Conclusion:**

We observe that MCI patients had higher gut dysbiosis than HC. However, despite increased dysbiosis, iNOS‐KO may preserve CBF and mitigate AD‐like symptoms, highlighting its potential neuroprotective role in the 3xTg‐AD model. Future studies should investigate the impact of iNOS‐KO on mitigating AD pathology, such as amyloid‐β and tau accumulation, or preserving cognitive functions. Our preliminary data shows that iNOS could be a potential target to ameliorate AD risk.